# Natural killer (NK) cell receptor-HLA ligand genotype combinations associated with protection from HIV infection: investigation of how protective genotypes influence anti HIV NK cell functions

**DOI:** 10.1186/s12981-017-0172-9

**Published:** 2017-09-12

**Authors:** Nicole F. Bernard

**Affiliations:** 1 Research Institute of the McGill University Health Centre (RI-MUHC), Montreal, QC Canada; 2 Division of Experimental Medicine, McGill University, Montreal, QC Canada; 3 Chronic Viral Illness Service, MUHC, Montreal, QC Canada; 4 Division of Clinical Immunology, MUHC, Montreal, QC Canada

**Keywords:** HIV exposed seronegative subjects, HIV susceptible subjects, NK cells, Killer-immunoglobulin-like receptor, HLA, Correlates of protection

## Abstract

The anti-HIV activity of natural killer (NK) cells could be induced fast enough to potentially prevent the establishment of HIV infection. Epidemiological studies identified two genotypes encoding NK receptors that contribute to NK cell function, that were more frequent in people who remained uninfected despite multiple HIV exposures than in HIV-susceptible subjects. NK cells from carriers of the *h/*y+B*57 genotype have higher NK cell functional potential and inhibit HIV replication in autologous HIV-infected CD4+ T cells (iCD4) more potently than those from carriers of non-protective genotypes. HIV suppression depends on the secretion of CC-chemokines that block HIV entry into CD4+ cells. NK cell education and the effect of HIV infection on iCD4 cell surface expression of MHC-I antigens both influenced NK cell responses to autologous iCD4. The second *KIR3DS1* homozygous protective genotype encodes an activating receptor that upon interacting with its HLA-F ligand on iCD4 induces anti-viral activity.

## Background

Some individuals remain uninfected despite high levels of exposure to HIV. Persons with this profile are called HIV exposed seronegative subjects (HESN) [1]. Understanding why HESN are resistant to HIV infection has the potential to identify correlates of protection from infection. As early as 2003, Scott-Algara et al. found that stimulating NK cells from HESN enrolled in a Vietnamese injection drug user cohort led to activation of higher levels of cytolysis and cytokine/chemokine secretion than did stimulating NK cells from HIV-infected persons or from seroconverters even before they seroconverted [2]. This was the first report suggesting that HESN may have NK cells with superior functionality.

Natural killer cells are important in innate host defences. They function in early responses to transformed and virus-infected cells, including to HIV-infected CD4+ T cells (iCD4) [3]. They express a plethora of cell surface receptors whose engagement initiate signals that culminate in inhibition or activation of effector functions. NK cell activity is regulated by the integration of signals from these receptors. An important family of NK receptors (NKR) are the killer immunoglobulin-like receptors (KIR). Their inhibitory counterparts (iKIR) use a subset of major histocompatibility complex class I (MHC-I) antigens as ligands. iKIR/HLA engagement educate developing NK cells for functionality and suppress the function of mature NK cells so that they are tolerant to normal self cells expressing MHC-I antigens [4]. HIV infected cells downmodulate HLA-A, -B and -C using Nef- and Vpu-mediated mechanisms and upregulate ligands for activating NKRs (aNKR) [5, 6]. This leads to NK cell activation of anti-HIV functions. NK cells express pre-stored IFN-γ transcripts, granzyme and perforin and are ready to lyse targets within minutes of activation [7]. They also secrete CCL3, CCL4 and CCL5, which bind and block CCR5, the co-receptor for HIV entry into CD4+ T cells [8].

Epidemiological studies reported that certain *KIR/HLA* genotypes were associated with slower time to AIDS in HIV-infected individuals implicating NK cells in HIV control [9, 10]. NK cells from individuals carrying the KIR3DS1/Bw4*80I combination, associated with slow progression to AIDS, were superior to those from carriers of either the receptor or ligand alone, in suppressing HIV replication in vitro [11]. The frequency of *KIR/HLA* genotypes in HESN and in HIV susceptible subjects enrolled in an HIV primary infection cohort was compared to address whether NK cells also play a role at the level of protection from HIV infection. HESN had higher frequencies of a high expression *KIR3DL1* homozygous (hmz) genotype termed KIR3DL1*h/*y co-carried with *HLA*-B*57 (*h/*y+B*57) and the *KIR3DS1*hmz genotype [12, 13]. A survival analysis performed on longitudinally followed HESN and HIV seroconverters found that time to seroconversion was significantly longer in individuals with, than without, these protective genotypes [14].

## Functionality of NK cells from carriers of *h/*y+B*57 genotypes

HLA-null cell stimulation of NK cells reveals their functional potential, which is directly related to how potently they were educated during development [15]. HLA-null stimulation of NK cells from *h/*y+B*57 carriers generated higher frequencies of NK cells expressing CD107 (a marker for degranulation) and secreting IFN-γ and TNF-α than NK cells from carriers of other *KIR3DL1*hmz/*HLA*-*B* genotypes [16, 17]. These triple functional cells exhibited a higher intensity of each of these functions than their mono-functional counterparts [17]. Thus, the *h/*y+B*57 genotype encoded iKIR receptor/HLA-B*57 ligand combinations that were particularly potent educating pairs that supported the development of NK cells with high functional activity. NK cells from *h/*y+B*57 carriers inhibited the replication of autologous iCD4 cells more potently than those from carriers of non-protective *KIR/HLA* genotypes [18]. Induction of NK cell inhibition of HIV replication was dependent on contact with autologous iCD4s. However, once NK cells were activated, their secreted products were sufficient to inhibit HIV replication. Among these, were the CC-chemokines CCL3, CCL4 and CCL5 detected in NK/iCD4 co-culture supernatants as well as by intra-cellular NK cell staining. Levels of CC-chemokines were highest in NK cells from *h/*y+B*57 carriers and higher in stimulated KIR3DL1+ than KIR3DL1− NK cells [18]. Neutralization of all three CC-chemokines in NK/iCD4+ co-cultures reversed inhibition of HIV replication [18]. Thus, the ability of NK cells to inhibit HIV replication is, at least in part, due to their ability to secrete CC-chemokines that block HIV infection of CD4+ T cells [8].

## Determinants of NK cell responsiveness to autologous iCD4

Natural killer cells respond to stimulation with autologous iCD4. By using fluorochrome conjugated antibody panels it is possible to gate on NK cell populations expressing defined inhibitory NKRs (iNKR) and to detect anti-viral functions such as secretion of IFN-γ/CCL4 and expression of CD107a by flow cytometry. This strategy has been used to explore the factors governing how NK cells from HIV-uninfected persons respond to their first encounter with autologous iCD4.

NKG2A/CD94 is an iNKR expressed on almost all CD56^bright^ and on ~40% of CD56^dim^ NK cells. The ligand for NKG2A in HLA-E, a non-classical MHC-Ib antigen whose cell surface expression depends on binding highly conserved peptides from the leader sequence of many HLA-A, B, C and G MHC-I molecules [19]. HLA-E is expressed on many cell types and maintained on iCD4 [20]. As NKG2A is an iNKR, the interaction of NKG2A with HLA-E on iCD4 should inhibit NK cell activation. However, the highest frequency of functional NK cells induced by autologous iCD4 were NKG2A+ NK cells within both the CD56^bright^ and CD56^dim^ compartments [21]. This NKG2A+ NK cell activation occurs because HLA-E on iCD4 presents an invariant HIV Gag derived peptide that abrogates NKG2A recognition and prevents negative signalling through this receptor [22].

KIR3DL1+ NK cells are educated in HLA-Bw4 donors and remain uneducated in HLA-Bw6 individuals expressing no HLA-Bw4 antigens. Autologous iCD4 triggered higher frequencies of responsive KIR3DL1+ NK cells from Bw4 than Bw6 donors [21, 23]. The frequency of responsive KIR3DL1+ NK cells was higher when from subjects carrying 1 copy than 2 copies of *HLA*-*Bw4* [23]. This finding is consistent with the ability of HIV Nef to downmodulate co-dominantly expressed HLA antigens on iCD4 to a threshold low enough to interrupt negative signaling through KIR3DL1. It may be easier to achieve this threshold in *Bw4/Bw6* heterozygotes encoding half the number of HLA-Bw4 antigens than do *Bw4*hmz [5, 24]. It is notable that secretion of CCL4, which has the potential to limit HIV infection, was the main contributor to total NK cell responsiveness to iCD4 [8, 21, 23].

HLA-C antigens can be dichotomized into C1 and C2 sub-groups. The HIV isolate used to infect CD4 cells in these studies retained cell surface HLA-C, making it available for interaction with KIR2DL3 on NK cells educated through this receptor, the consequence of which is to inhibit KIR2DL3+ NK cell activation. KIR2DL3+ NK cells are educated in HLA-C1 donors but remain largely uneducated in *HLA*-*C2*hmz [25]. The frequency of KIR2DL3+ NK cells from *C1*hmz responding to autologous iCD4 was lower than that of *C2*hmz. As for KIR3DL1+ NK cells, a ligand dosage effect was observed where iCD4 from *C1*hmz suppressed KIR2DL3+ NK cell function more potently than did those from *C1/C2* heterozygotes. The 2 copies of C1 ligand expressed on iCD4 from *C1*hmz likely engaged KIR2DL3 more potently that the CI expressed on iCD4 from *C1/C2* heterozygotes, resulting in more potent inhibition of KIR2DL3+ NK cell function [23]. HLA-C is expressed on the cell surface at lower levels than HLA-A and -B antigens [26]. This may also contribute to the less potent KIR2DL3+ NK cell inhibitory capacity of iCD4 from *C1/C2* carriers than *C1*hmz subjects. Secretion of CCL4 was the main contributor to the total KIR2DL3+ NK cell responsiveness to iCD4.

## Functionality of KIR3DS1+ NK cells from KIR3DS1hmz

The *KIR3DS1*hmz genotype is also associated with protection from HIV infection [13, 14]. The notion that the ligand for KIR3DS1 was a subset of Bw4 antigens with an isoleucine at position 80 of the HLA heavy chain (Bw4*80I) has recently been debunked in favor the non-classical MHC-Ib antigen, HLA-F [27]. HLA-F is expressed on the HLA-null 721.221 and K562 cells and on HIV-infected cells [27].

The KIR genetic region is not only polymorphic but also polygenic. Next generation sequencing of this region showed that genes in the centromeric and telomeric KIR regions are in linkage disequilibrium with each other and separated by a recombination hotspot [28]. *KIR3DS1* lies in a telomeric KIR B haplotype region know as TB01. When *KIR3DS1* is present so is the linked adjacent gene, *KIR2DL5* [28]. The linkage disequilibrium (LD) between the 2 genes raises the possibility that genes in LD with KIR3DS1 are responsible for the observed association of the *KIR3DS1*hmz genotype with HIV resistance observed in HESN. Although the KIR genes in the TB01 region generally segregate together, their gene products are stochastically expressed in NK cell populations. When NK cells from *KIR3DS1*hmz expressing all possible combinations of KIR3DS1 and KIR2DL5 were stimulated with HLA-F+ 721.221 HLA-null cells, only the NK cell populations expressing KIR3DS1 responded above background levels while expression of KIR2DL5 made no contribution (Jackson et al., submitted). Thus, KIR3DS1+ NK cells are functional and activatable by HLA-F+ cells. Further investigation is needed to confirm that these NK cells also respond to autologous iCD4 and that responsiveness can be ascribed to the interaction of KIR3DS1 with HLA-F on iCD4.

## Conclusions

Genotypes encoding NKRs that are overrepresented in HESN confer NK cells with superior functional potential to HLA-null cells and with superior responses to autologous iCD4. NK cell education is important in the responsiveness of NK cell populations to iCD4. The higher functionality, in terms of CCL4 secretion, of educated KIR3DL1+ NK cells may be a mechanism underlying the superior viral control and slower time to AIDS observed in epidemiological studies for some carriers of *KIR3DL1*hmz/*Bw4* compared to carriers of other *KIR/HLA* genotypes [10]. The functionality of NK cell populations to autologous iCD4 is not only dependent on their education but also on HIV-mediated changes in MHC-I expression levels. An important component of the anti-viral activity of stimulated NK cells is secretion of CC-chemokine, which can block HIV entry into new CD4+ target cells.

That NK cells can play a role at the level of protection from HIV infection and in slowing HIV disease course prompts a consideration of how to harness their anti-HIV activity for preventative and therapeutic vaccine development. It may be possible to activate NK cells for HIV directed effector functions by using vaccines that induce anti-HIV specific antibodies whose Fc portions interact with the aNKR CD16. Mechanisms such as this one are likely what accounted for the success of the RV144 Thai HIV vaccine trial in preventing HIV infection [29, 30]. Another avenue to explore that is in its infancy at present, is to find ways to induce memory-like NK cells that may be able to respond faster and more potently to HIV exposure than conventional NK cells [31].
